# Inhomogeneous Sparseness Leads to Dynamic Instability During Sequence Memory Recall in a Recurrent Neural Network Model

**DOI:** 10.1186/2190-8567-3-8

**Published:** 2013-07-22

**Authors:** Daniel Medina, Christian Leibold

**Affiliations:** 1 Department Biologie II, Ludwig-Maximilians-Universität, Munich, Germany; 2 Bernstein Center for Computational Neuroscience, Munich, Germany

**Keywords:** Associative memory, Sequence memory, Memory capacity, Sparse coding

## Abstract

Theoretical models of associative memory generally assume most of their parameters to be homogeneous across the network. Conversely, biological neural networks exhibit high variability of structural as well as activity parameters. In this paper, we extend the classical clipped learning rule by Willshaw to networks with inhomogeneous sparseness, i.e., the number of active neurons may vary across memory items. We evaluate this learning rule for sequence memory networks with instantaneous feedback inhibition and show that little surprisingly, memory capacity degrades with increased variability in sparseness. The loss of capacity, however, is very small for short sequences of less than about 10 associations. Most interestingly, we further show that, due to feedback inhibition, too large patterns are much less detrimental for memory capacity than too small patterns.

## 1 Introduction

Many brain areas exhibit extensive recurrent connectivity. Over decades such neuronal feedback attracted a huge amount of theoretical modeling [1-3] and one of the most prominent functions that is proposed for the recurrent synaptic connections is that of associative memory. In most such theories, all memory items are generally treated as equal, particularly in terms of the sparseness with which they are neurally represented, i.e., in terms of how many neurons are active during recall. In this paper, we extend a particular class of such auto-association networks, viz., sequence memory networks, to include variable sparseness and thereby add one aspect of variability that is to be expected in biological neural networks. 

Memory sequences have been shown to occur in the rodent brain during hippocampal sharp-wave ripple events [4,5]. The major hypothesis of the present paper is that these sequences are stored in the recurrent connections of the hippocampal network, which is supported by findings of fast coordinated excitatory synaptic currents during sharp-wave ripple events in slices [6]. 

Here, we build on a previous model of memory sequences [7], which enhances memory capacity by instantaneous feedback inhibition. Both our mean field analysis and our simulations show that, in this model, inhomogeneity in pattern sizes reduces memory capacity, but it does so in an asymmetric way: whereas too small patterns significantly compromise the stability of sequence recall, too large patterns can be compensated for quite robustly. 

## 2 Model

We use the standard formalism of auto-associative networks: a discrete-time dynamical system. The individual time steps may be interpreted as the cycles of the hippocampal ripple oscillations. The states $x_{i}(t)$ of all neurons 1≤i≤N at time step *t* determine the states at time t+1 by a thresholded function

(1) $$x_{i}(t+1)=\Theta \left(\sum_{j=1}^{N}J_{ij}x_{j}(t)-\theta \right).$$

Here, as in many other approaches, we define *Θ* as the Heaviside function, which is equivalent to restricting the neuron states to binary variables, with $x_{i}=1$ if neuron *i* fires and $x_{i}=0$ if it is silent. The state of the network at time *t* is thus denoted by $x(t)\in {\left\{0,1\right\}}^{N}$. The other parameters are the firing threshold *θ* and the synaptic weights $J_{ij}$.

In the framework of the dynamical system of (1), associative memory is considered to be the (approximate) recall of a network state $x(t+n)=\xi_{t+n}$ at time t+n after the network has been initialized with some appropriate cue $x(t)=\xi_{t}$ at time *t*. Recall can either occur as convergence to a dynamical attractor (n→∞) [2], or as a one-step association (n=1) [8,9]. The specific choice of the synaptic matrix $J_{ij}$ determines which patterns can be recalled, or *are stored* in the network.

In this paper, we will deal with sequences of one-step associations with binary synapses [8,10,11] and instantaneous feedback inhibition [7,9,12]. Here, memory sequences are described as sequences of random activity patterns *ξ* that are binary vectors of dimension *N*, $\xi \in {\left\{0,1\right\}}^{N}$. A memory sequence of length *Q* is an ordered occurrence of activity patterns $\xi_{1}\to \xi_{2}\to \cdots \to \xi_{Q}$. The number $M_{t}$ of active neurons in each pattern $\xi_{t}$ is called pattern size, and will in general be different for each pattern.

As proposed in [7,9,11], we model the weights $J_{ij}$ of the binary synapses as products of two independent binary stochastic processes, $J_{ij}=w_{ij}s_{ij}$. The first stochastic variable $w_{ij}$ indicates the presence ($w_{ij}=1$) or absence ($w_{ij}=0$) of a morphological connection, with probability $\mathrm{Pr}(w_{ij}=1)=c_{m}$, called *morphological connectivity*. The second process $s_{ij}$ is called synaptic state and will be used to store memories. In the potentiated state ($s_{ij}=1$), a presynaptic spike increments the postsynaptic potential by 1, whereas in the silent state ($s_{ij}=0$), the postsynaptic potential remains unaffected. According to (1), neuron *i* fires a spike at cycle t+1 if its postsynaptic potential $h_{i}(t)=\sum_{j=1}^{N}w_{ij}s_{ij}x_{j}(t)$ at time *t* exceeds the threshold *θ*.

Willshaw’s [10] clipped Hebbian rule is used to set the synaptic states $s_{ij}$ such that the network is able to recall the memory sequences: a synapse is in the potentiated state only if it connects two neurons that are activated in sequence at least once.

In the case of homogeneous sparseness, where all patterns have the same number *M* of active neurons, Willshaw’s rule connects the fraction $c/c_{m}$ of potentiated synapses in the network with the number *P* of stored associations by

(2) $$P=\frac{\log(1-c/c_{m})}{\log(1-f^{2})}$$

where f=M/N is the coding ratio. The effective connectivity *c* defines the noise level during recall, i.e., how many spurious inputs a neuron gets that are not part of the memory pattern to be recalled. If *c* is too large, the network will exhibit many spurious activations and the memory can no longer be recalled. Equation (2) thus provides a capacity estimate of the network in that it says how many associations are stored at the maximum noise level *c*.

**Fig. 1:**
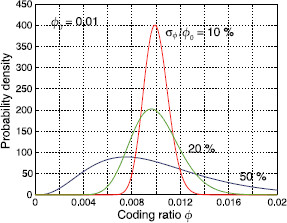
Coding ratio distribution $p_{\phi }(\phi )$. We use the Gamma distribution in our simulations, shown here for a mean coding ratio $\phi_{0}=0.01$ and three different values of the variation coefficient $\sigma_{\phi }/\phi_{0}$

In the case of inhomogeneous sparseness, this formula is no longer valid and thus we asked what would its generalization be. To this end, we introduce the coding ratio vector

(3) $$\phi =\{f_{0},f_{1},f_{2},\ldots ,f_{P}\}$$

where $M_{k}=f_{k}N$ is the number of active neurons in the stored pattern $\xi_{k}$. The elements of ***ϕ*** are considered to be random variables and distributed according to the coding ratio distribution $p_{\phi }(\phi )$, with mean coding ratio $\phi_{0}$ and standard deviation $\sigma_{\phi }$. For now, we use a Gamma distribution (Fig. 1) for $p_{\phi }(\phi )$ with mean $\phi_{0}=0.01$. By varying the standard deviation $\sigma_{\phi }$ we control how much the elements of vector ***ϕ*** deviate from its mean $\phi_{0}$. 

In analogy to (2), the probability of synaptic potentiation $\varsigma =c/c_{m}$ can be computed analytically for any given coding ratio vector ***ϕ*** (see the Appendix) as follows:

(4) $$\varsigma =1-\prod_{k=1}^{P}(1-f_{k}f_{k-1}).$$

**Fig. 2:**
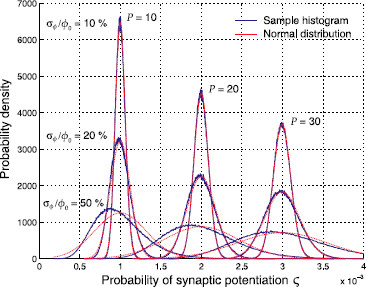
Distribution of the synaptic potentiation probability *ς* over all possible realizations of the coding ratio vector ***ϕ*** for three different values of the number of patterns *P* and the variation coefficient $\sigma_{\phi }/\phi_{0}$. The histograms (*blue*) correspond to one million random samples of the coding ratio vector ***ϕ***. The elements of ***ϕ*** are drawn randomly from the Gamma distribution with $\phi_{0}=0.01$, shown in Fig. 1. The normal distribution (*red*) with mean 〈ς〉 and variance $\sigma_{\varsigma }^{2}$ (see the Appendix) is a good approximation to the true distribution of *ς* for low values of $\sigma_{\phi }/\phi_{0}$

This expression, however, only provides an implicit dependence on the number *P* of patterns, and moreover, it of course depends on the specific choice of ***ϕ***. We therefore asked how much *ς* varies over the statistical ensemble of ***ϕ***. In addition to addressing this question numerically, we could also find analytical expressions for the mean 〈ς〉 and variance $\sigma_{\varsigma }^{2}$ of *ς* over all possible realizations of ***ϕ***, which depend on the first to fourth-order moments of the coding ratio distribution $p_{\phi }(\phi )$ (see the Appendix). For sufficiently small variances $\sigma_{\phi }^{2}$, the Gaussian approximations resulting from 〈ς〉 and $\sigma_{\varsigma }^{2}$ fit the empirical distributions of *ς* very well; see Fig. 2 for 10^6^ random samples of ***ϕ***. 

The results show that the variability in *ς* is actually relatively large (about 10 % for $\sigma_{\phi }=0.1\phi_{0}$) and even increases with increasing number of associations *P*. We therefore decided not to use the expectation value 〈ς〉 for further discussion, but to show empirical distributions of many realizations of ***ϕ*** whenever possible.

In order to evaluate the dynamics of sequence retrieval, we mostly do not simulate the full neural network but use a mean field approximation [7,11] based on two macroscopic dynamic variables: the number $m_{t}\in [0,M_{t}]$ of correctly activated neurons (*hits*) and the number $n_{t}\in [0,N-M_{t}]$ of incorrectly activated neurons (*false alarms*). For large network sizes *N* and large pattern sizes $M_{t}$, the central limit theorem predicts the distribution of the total number of synaptic inputs h(t) to be Gaussian, and the variables $m_{t}$ and $n_{t}$ can be reinterpreted as expectation values over many realizations of the connectivity matrix. Denoting the mean number of synaptic inputs as μ≡〈h(t)〉 and the variance as $\sigma^{2}\equiv \langle h{\left(t\right)}^{2}\rangle -{\langle h(t)\rangle }^{2}$, we obtain (see the Appendix) for the **On** population (should fire),

(5) $$\mu_{\mathrm{On}}=c_{m}m_{t}+c_{m}\varsigma n_{t},$$

(6) $$\sigma_{\mathrm{On}}^{2}=c_{m}m_{t}(1-c_{m})+c_{m}\varsigma n_{t}\left(1-c_{m}\varsigma +V_{\varsigma }^{2}c_{m}\varsigma (n_{t}-1)\right)$$

and for the **Off** population (should not fire),

(7) $$\mu_{\mathrm{Off}}=c_{m}\varsigma (m_{t}+n_{t}),$$

(8) $$\sigma_{\mathrm{Off}}^{2}=c_{m}\varsigma (m_{t}+n_{t})\left(1-c_{m}\varsigma +V_{\varsigma }^{2}c_{m}\varsigma (m_{t}+n_{t}-1)\right).$$

Willshaw’s learning rule yields correlations in the synaptic states that are captured by the terms proportional to $V_{\varsigma }^{2}$ (see the Appendix).

The discrete-time network dynamics in (1) maps to the mean field model such that

(9) $$(m_{t+1},n_{t+1})=\left(T_{\mathrm{On}}(m_{t},n_{t}),T_{\mathrm{Off}}(m_{t},n_{t})\right)$$

with

(10) $$T_{\mathrm{On}}(m_{t},n_{t})=M_{t+1}\Phi \left(\frac{\mu_{\mathrm{On}}-\theta }{\sigma_{\mathrm{On}}}\right),$$

(11) $$T_{\mathrm{Off}}(m_{t},n_{t})=(N-M_{t+1})\Phi \left(\frac{\mu_{\mathrm{Off}}-\theta }{\sigma_{\mathrm{Off}}}\right)$$

where $\Phi (z)\equiv [1+\mathrm{erf}(z/\sqrt{2})]/2$ denotes the cumulative distribution function (cdf) of the normal distribution.

In the framework of this model and following [7,12], inhibition is introduced as instantaneous negative feedback proportional to the total number $m_{t}+n_{t}$ of active neurons at time *t*. Formally, this is achieved by substituting $\theta \to \theta +h_{t}$ in (10) and (11), where $h_{t}=b(m_{t}+n_{t})$. The inhibitory weight is taken as $b=c_{m}\varsigma$ throughout the paper (for discussion see [7]). 

## 3 Results

### 3.1 Inhomogeneous Sparseness Reduces Dynamic Stability

**Fig. 3:**
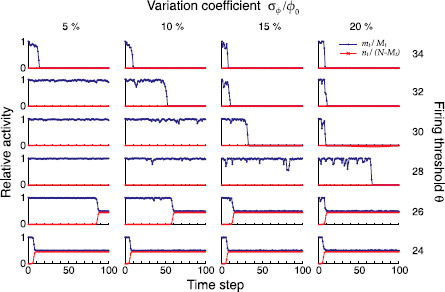
Inhomogeneous pattern sizes lead to dynamic instability during sequence replay. In all graphs, we show the fraction $m_{t}/M_{t}$ of hits (*blue*) at time step *t* and the fraction $n_{t}/(N-M_{t})$ of false alarms (*red*) during the replay of a sequence of length Q=100, using the mean field model of equations (9) and following. Left to right: increasing inhomogeneity $\sigma_{\phi }$. Bottom to top: increasing firing thresholds *θ*. Other parameters were $N=10^{5}$, $c_{m}=0.1$, c=0.05 and $\phi_{0}=0.01$

Some exemplary numerical evaluations of the mean field dynamics from equations (9) and following for different firing thresholds *θ* and different pattern size inhomogeneities $\sigma_{\phi }$ are shown in Fig. 3. Despite being just random samples, these plots already reveal the impact of increasing inhomogeneity in the coding ratio vector ***ϕ*** on the network’s ability to successfully retrieve the stored patterns. As the standard deviation $\sigma_{\phi }$ grows, the activity fluctuations during replay become more and more pronounced and, at some point, lead to dynamic instability during recall. This is clearly visible for a firing threshold θ=28, where perfect pattern retrieval ($m_{t}/M_{t}=1$ and $n_{t}/(N-M_{t})=0$) is interrupted more and more frequently as $\sigma_{\phi }$ increases, eventually preventing the retrieval of the full sequence at $\sigma_{\phi }/\phi_{0}=20\;\%$. There the network falls silent, due to a small pattern in the sequence that no longer generated the required synaptic drive to retrieve the following pattern in the sequence. For lower thresholds (θ=24,26), the network activity may instead explode prematurely due to a big pattern that generates too much synaptic drive and sets the network into a permanently active (epileptic) state. As we simulate a network with instantaneous feedback inhibition, the epileptic state is characterized by approximately half of the neurons being active at any time ($m_{t}/M_{t}\approx n_{t}/(N-M_{t})\approx 1/2$), where the subset of active neurons changes from time step to time step. 

In summary, the range of thresholds under which the network successfully replays the full sequence is severely reduced as the pattern sizes become more and more inhomogeneous.

#### 3.1.1 Replay Success Rate

In order to analyze the numerical results more quantitatively, we introduce a criterion for what we consider to be a successful replay. Following [11], we define the retrieval quality

(12) $$\Gamma_{t}\equiv m_{t}/M_{t}-n_{t}/(N-M_{t})$$

as the relative difference between hit ratio and false alarm ratio, and consider a pattern at time *t* to be retrieved successfully if $\Gamma_{t}>0.5$. By running the mean field equations many times with different random realizations of vector ***ϕ***, we obtain an empirical *replay success rate*$\varrho_{t}$ as the fraction of runs with successful retrieval $\Gamma_{t}>0.5$.

**Fig. 4:**
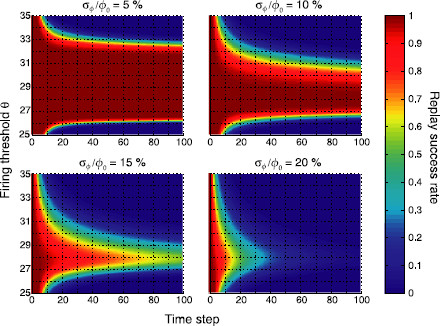
Replay success rate *ϱ* over time *t* for different firing thresholds *θ* and a sequence of length Q=100. *Panels* show different levels of inhomogeneity ($\sigma_{\phi }$). Parameters were $N=10^{5}$, $c_{m}=0.1$, c=0.05 and $\phi_{0}=0.01$ leading to P≈7000 stored patterns

Figure 4 shows replay success rates for a sequence of length Q=100 for $\phi_{0}=0.01$ and varying inhomogeneities $\sigma_{\phi }$. When the *P* stored associations are still approximately homogeneous (*top left*), the full sequence can be retrieved with probability one for a large range of firing thresholds *θ*. As we let inhomogeneity increase, this range becomes narrower (*top right*), and eventually collapses (*bottom*), so that only the first items can be retrieved with high probability. Hence, inhomogeneous sparseness strongly affects the replay of long sequences, but does relatively little harm to short sequences (Q⪅5). 

#### 3.1.2 Region of Stable Replay

**Fig. 5:**
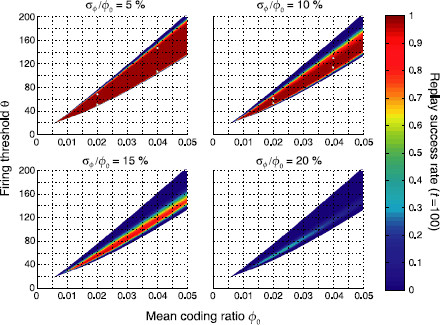
Regions of stable sequence replay in $\left(\phi_{0},\theta \right)$ space. *Colors* indicate the replay success rate at time step t=100. *The white area* corresponds to the region of no retrieval for homogeneous pattern sizes ($\sigma_{\phi }=0\;\%$). *Panels* show different levels of inhomogeneity ($\sigma_{\phi }$). The region of retrieval obtained from the mean field equations was validated with computer simulations of the corresponding networks of binary neurons (*white discs*, 95 % success rate). Parameters were $N=10^{5}$, $c_{m}=0.1$, and c=0.05

Sequence retrieval not only critically depends on the firing threshold *θ*, but also on the mean coding ratio $\phi_{0}$. We therefore searched for regions of stable replay in $\left(\phi_{0},\theta \right)$ space (Fig. 5) at time step t=100. The region where sequence replay for homogeneous patterns ($\sigma_{\phi }=0$) is unfeasible is shown in white for comparison. Replay regions exhibit the typical wedge shape [7,11]. If the firing threshold *θ* is too low or the mean coding ratio $\phi_{0}$ is too large, all neurons immediately start to fire and the network falls into an all-active state. If the firing threshold is too high or the mean coding ratio is too small, the network immediately falls into an all-silent state. As patterns become less homogeneous, the wedge-shaped region of replay becomes narrower and narrower, and eventually vanishes for highly inhomogeneous patterns (*bottom right*). The region of retrieval obtained from the mean field equations was validated with some computer simulations of the corresponding networks of binary neurons (*white discs*, corresponding to 95 % replay success rate). 

### 3.2 Storage Capacity

**Fig. 6:**
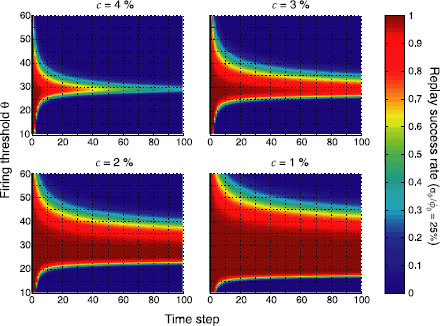
Replay success rate for a variation coefficient of $\sigma_{\phi }/\phi_{0}=25\;\%$; cf. Fig. 4. *Panels* show results for different mean connectivities *c*, and hence different numbers *P* of stored associations

As in the classical Willshaw net ([10], and (2)) the mean synaptic connectivity *c* for a network with inhomogeneous pattern sizes depends on the number *P* of stored associations, as well; see (4). This allowed us to adjust the mean connectivity to a fixed value c=0.05 by changing the number *P* of stored associations in the network. So far, we have kept *c* constant and varied the width parameter $\sigma_{\phi }$ of the size distribution. We saw that for large inhomogeneities ($\sigma_{\phi }/\phi_{0}⪆20\;\%$) replay of long sequences is hardly possible. But what if we reduce *c*? Intuitively, replay should become more stable if we reduce the “noise” connectivity *c*. As shown in Fig. 6, this is indeed the case: If the number of stored associations, and thus *c*, is decreased, sequence retrieval is robust under high inhomogeneity, and may even allow for replay of the full sequence for a whole range of firing thresholds. 

However, reducing *c* comes at the cost of a reduced memory capacity *P*, and thus we have to find a way to quantify the trade-off between replay stability and capacity for a network with inhomogeneous patterns sizes. To this end, we define the *maximum retrievable sequence length*

(13) $$T(\phi ,c_{m})=\max_{\theta }T_{90}(\phi ,c_{m},\theta )$$

as the maximum number $T_{90}$ of time steps for which the replay success rate $\varrho_{T}$ remains above 90 % for a given pattern size vector ***ϕ*** and morphological connectivity $c_{m}$. Since replay stability strongly depends on the firing threshold, the maximum is taken over all possible firing thresholds *θ*.

**Fig. 7:**
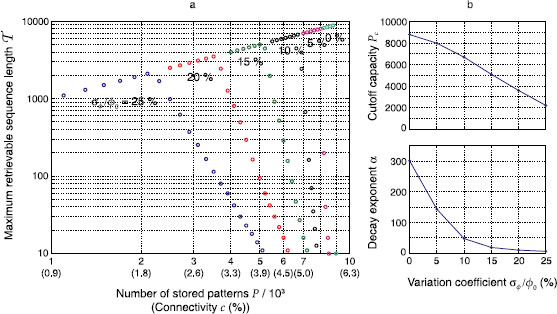
Inhomogeneity reduces memory capacity. **a** The maximum retrievable sequence length  is shown (*colored discs*) as a function of the number *P* of associations stored in the network and the corresponding mean connectivity *c*. *Different colors* indicate inhomogeneities ($\sigma_{\phi }/\phi_{0}$). **b** Power law fits to the decreasing parts of the graphs in **a** give rise to a cutoff capacity $P_{c}$ (*top*) at which the curves start to fall, and an exponent *α* (negative slopes in **a**) of the power law decrease. Parameters were $N=10^{5}$, $c_{m}=0.1$, and $\phi_{0}=0.01$

Figure 7 shows  as a function of the number *P* of stored associations in the network (as well as the corresponding mean connectivity *c*). When the total number of stored associations is small, even a sequence consisting of all stored associations may be retrieved under high inhomogeneity ($\sigma_{\phi }/\phi_{0}=25\;\%$). As *P* grows, the  curve reaches a maximum and then decreases very quickly. The breakdown point and slope depend critically on the degree of inhomogeneity. For the homogeneous case $\sigma_{\phi }\to 0$,  decreases infinitely steeply, and the number *P* of patterns at the breakdown determines the “classical” storage capacity. For finite inhomogeneities $\sigma_{\phi }$,  decreases according to a power law $T\propto P^{-\alpha }$ that trades stability  vs. capacity *P*. Since the exponent *α* is much smaller for high inhomogeneities $\sigma_{\phi }/\phi_{0}$, the net decrease of capacity for short sequences (⪅10) is relatively small compared to a network with homogeneous sparseness (a decrease of ∼1.8 for $\sigma_{\phi }/\phi_{0}=25\;\%$). 

### 3.3 Asymmetry of the Size Distribution

From our observations of single runs in Fig. 3, we already derived some anecdotal insight into the mechanisms underlying the breakdown of sequence replay: network activity may cease after a small pattern, whereas large patterns may lead to epilepsy. However, it is unclear which of these two ways of terminating replay is more problematic, or whether both occur equally often.

**Fig. 8:**
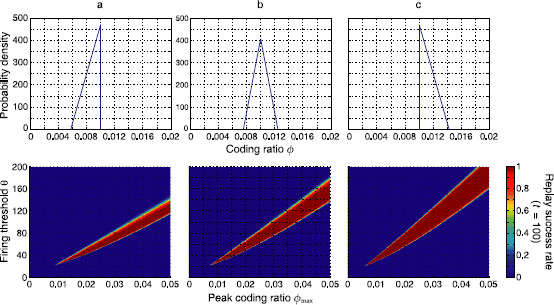
Mechanisms of sequence termination. *Top*: three triangular coding ratio distributions $p_{\phi }(\phi )$ with maximum at $\phi_{\max}=0.01$, mean coding ratios $\phi_{0}=\phi_{\max}$ ($\pm \sqrt{2}\sigma_{\phi }$), and fixed standard deviation $\sigma_{\phi }=0.1\phi_{\max}$: **a** negatively skewed distribution, **b** symmetric distribution, **c** positively skewed distribution. *Panels* (cf. Fig. 5): Replay success rates in the $\left(\phi_{\max},\theta \right)$ plane. The region of retrieval obtained from the mean field equations was validated with computer simulations of some of the corresponding networks of binary neurons (*white discs*, 95 % success rate). Parameters were $N=10^{5}$, $c_{m}=0.1$, and c=0.05

In order to tackle this question about the mechanisms of sequence termination, we investigate the effect of *skewness* (or asymmetry) in the pattern size distribution, i.e., an imbalance between bigger and smaller than average patterns. So far, we have used the Gamma distribution shown in Fig. 1, which is relatively symmetric for small variation coefficients. To have a better handle on skewness, we now switch to triangular distributions (Fig. 8). 

A symmetric triangular distribution (Fig. 8b) is used for comparison. In order to study the effect of an excess of small patterns we constructed a negatively skewed distribution (Fig. 8a) by cutting away all patterns above the line of symmetry ($\phi_{\max}$) and adding smaller patterns instead. Since this distribution has a lower mean coding ratio $\phi_{0}=\phi_{\max}-\sqrt{2}\sigma_{\phi }$, we increased the number *P* of patterns to account for the same “noise” connectivity *c* as in the symmetric case. The region of stable replay is clearly reduced in the negatively skewed distribution as compared to the symmetric one. This reduction could either be because small pattern sizes are intrinsically bad, or because the asymmetry of the distribution is a limiting factor. We therefore also considered the case of an excess of large patterns (Fig. 8c). For such a positively skewed distribution, the asymmetry is the same as for the negatively skewed distribution; however, and interestingly, the region of stable replay is larger than for the symmetric distribution. Again, the connectivity was adjusted to the same value by reducing the number *P* of associations to compensate for the higher mean coding ratio $\phi_{0}=\phi_{\max}+\sqrt{2}\sigma_{\phi }$.

From these observations, we conclude that indeed the small patterns are much more problematic for replay with inhomogeneous pattern sizes than the large patterns. To understand why, we compared the shape of the replay regions for the three distributions (a, b, and c), and observe that the slope of the lower side of the wedge is relatively insensitive to skewness, whereas the slope of the upper side of the wedge is very different in each case. Failures owing to activity explosion (the lower side of the wedge) are almost independent of the skewness, due to the instantaneous feedback inhibition in the mean field equations. On the other hand, the upper side of the wedge is determined by the network’s falling into a silent state. Thus, positive skewness (c) is the more robust distribution. Note that this was also apparent in Fig. 5, where the reduction of the region of stability with increasing inhomogeneity was much more pronounced on the upper side of the wedges than on the lower side, despite the relatively symmetric Gamma distribution used there.

**Fig. 9:**
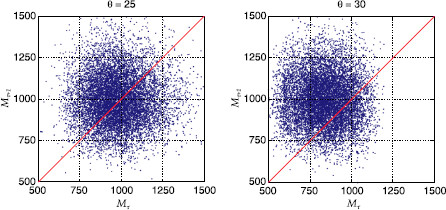
Scatter plot of pairs $\left(M_{\tau },M_{\tau +1}\right)$, where $\xi_{\tau }$ is the last correctly replayed pattern ($\Gamma_{\tau }>0.5$) in each sequence, for 10^4^ random samples of ***ϕ*** from the Gamma distribution and two different firing thresholds *θ* as indicated. Parameters were as in the third column of Fig. 4: $N=10^{5}$, $c_{m}=0.1$, c=0.05, $\phi_{0}=0.01$, and $\sigma_{\phi }/\phi_{0}=15\;\%$

Finally, in order to more directly illustrate how replay terminates after small patterns, we show a scatter plot of 10^4^ sample pairs $\left(M_{\tau },M_{\tau +1}\right)$, where *τ* is the last time step at which the pattern was replayed with sufficient quality $\Gamma_{\tau }>0.5$ (Fig. 9; note that here we again used a Gamma distribution to achieve comparability with Fig. 4). Points below the red line ($M_{\tau }>M_{\tau +1}$) represent sequences for which the last correctly replayed pattern $\xi_{\tau }$ was bigger than the following pattern $\xi_{\tau +1}$, whereas points above the red line ($M_{\tau }<M_{\tau +1}$) represent sequences for which $\xi_{\tau }$ was smaller than $\xi_{\tau +1}$. On the low-threshold edge of the stability region, which is prone to over-excitement (θ=25, cf. Fig. 5), a big-to-small pattern transition is as likely to lead to a failure in sequence replay as a small-to-big transition. However, on the high-threshold edge of the stability region (θ=30), where replay eventually dies out, most failures are caused by small-to-big pattern transitions (∼80% of points above the red line). 

Again, these results show that the small patterns are more detrimental for sequence replay than the large patterns, since in the latter case fluctuations can be compensated for by feedback inhibition, whereas the former have no compensatory mechanism.

### 3.4 Nonlinear Inhibition

So far, we have assumed a linear dependence of instantaneous feedback inhibition on the total network activity, i.e., $h_{t}=b(m_{t}+n_{t})$, since it was shown to optimize replay quality [7,12]. In this final section, we investigate how a particular *nonlinear* form of inhibition could improve the network’s resilience to inhomogeneity, because (a) physiological data from cortical inhibitory networks suggest supralinear dependence on input [13,14] and (b) supralinear inhibition effectively provides a positive feedback (with respect to linear inhibition) in cases of too low activity. 

**Fig. 10:**
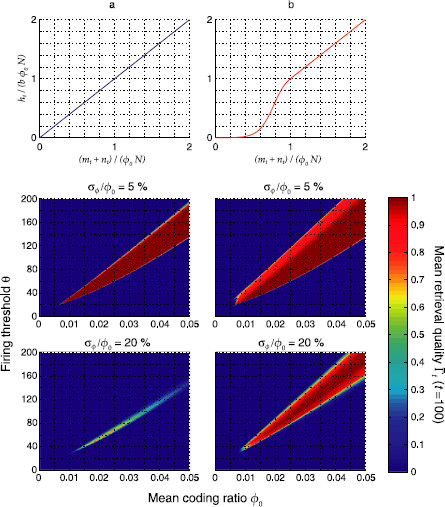
Regions of stable sequence replay in the $\left(\phi_{0},\theta \right)$ plane with linear (**a**) and nonlinear (**b**) feedback inhibition. *Colors* indicate the mean retrieval quality ${\bar{\Gamma }}_{t}$ at time step t=100. *Panels* show different levels of inhomogeneity ($\sigma_{\phi }$). The region of retrieval obtained from the mean field equations was validated with computer simulations of the corresponding networks of binary neurons (*white discs*, 95 % success rate). Parameters were $N=10^{5}$, $c_{m}=0.1$, and c=0.05

To be able to best compare the nonlinear with the linear case, we constructed a nonlinearity that only implements such positive boost for too low activities $m_{t}+n_{t}$ and remains linear for too large activities (see upper panel of Fig. 10b). Formally, it is obtained by replacing the linear term $b(m_{t}+n_{t})$ by the function $h_{t}=h(m_{t}+n_{t})$, where

(14) $$h(x)=\left\{ \begin{array}{ll} \frac{\kappa }{1+e^{-\lambda (x-\nu )}}, & x\leq \phi_{0}N,\\ bx, & x>\phi_{0}N \end{array}\right.$$

with parameters

(15) $$\kappa =\frac{b\lambda {\left(\phi_{0}N\right)}^{2}}{\lambda \phi_{0}N-1},$$

(16) $$\nu =\phi_{0}N-\frac{1}{\lambda }\log(\lambda \phi_{0}N-1),$$

(17) $$\lambda =10^{-4}/\phi_{0}$$

chosen such that the slope of h(x) at the operation point $x=m_{t}+n_{t}=\phi_{0}N$ is equal to *b* in both the linear and nonlinear parts. 

Figure 10 shows the mean retrieval quality ${\bar{\Gamma }}_{t}$ at time step t=100 (averaged over 10^2^ random realizations of vector ***ϕ***) in the $\left(\phi_{0},\theta \right)$ plane, for linear (a) and nonlinear (b) inhibition and two levels of inhomogeneity $\sigma_{f}/\phi_{0}$.

For low inhomogeneity ($\sigma_{\phi }/\phi_{0}=5\;\%$), although the region of stability is wider in the nonlinear case, the retrieval quality in the gained region is not as good as in the region shared by both feedback strategies (see lighter red stripe in middle panel of Fig. 10b). This finding fits very well to previous papers that report that linear inhibition maximizes replay quality for homogeneous pattern sizes: the gain in robustness is mostly obtained by a reduced replay quality. For a large inhomogeneity ($\sigma_{\phi }/\phi_{0}=20\;\%$), linear feedback almost completely extinguishes replay, whereas nonlinear inhibition recovers a considerable stable replay region with high retrieval quality.

Supralinear inhibitory feedback at low activity levels thus significantly widens the replay region making the network resilient to higher levels of inhomogeneity than would be possible with linear feedback. The underlying mechanism by which this is achieved can be explained as follows: smaller-than-average patterns generate only little negative feedback and thereby keep up the activity in the network, whereas bigger-than-average patterns are compensated for optimally by linear negative feedback.

## 4 Discussion

This paper extends previous models of sequence memory [7,9,11,12] that were based on Willshaw’s learning rule [10] to *inhomogeneous* pattern sizes, i.e., patterns of variable sparseness. Our work reveals that inhomogeneity in the sparseness of stored patterns is detrimental to a recurrent network’s dynamic stability during sequence retrieval. Bigger than average patterns tend to lead the network into an all-active epileptic state as a result of an excessively high synaptic drive, whereas smaller than average patterns tend to lead to an all-silent state as a result of an insufficient synaptic drive. In either case, sequence retrieval is terminated prematurely due to dynamic instability. As expected, the higher the variability in pattern sizes, the higher is the probability of premature sequence termination. Our results thus suggest that a plasticity mechanism that ensures a certain degree of homogeneity in the sparseness of hippocampal representations would be useful for the reliable retrieval of long sequences.

Instantaneous linear feedback inhibition is able to compensate to a certain degree for bigger-than-average patterns, but it does nothing to prevent the network from falling silent since it does not compensate for an insufficient synaptic drive. This asymmetry is reflected on the relative impact of differently skewed pattern size distributions. Compared to a symmetric distribution, negative skewness leads to a smaller region of stable replay, whereas positive skewness leads to a larger region. Positively skewed pattern size distributions are thus more resilient to premature sequence termination under linear feedback. The higher vulnerability to smaller-than-average patterns can be corrected for by introducing a nonlinear negative feedback which is close to zero for lower-than-average network activity. Such supralinear inhibition can make the network resilient to higher levels of inhomogeneity than linear feedback inhibition.

Memory networks with variable sparseness have been studied by Amit and Huang [15,16] under a different learning paradigm in which old memories are gradually overwritten by new memories, and for several more involved synaptic (meta-)plasticity rules. There, inhomogeneity in the pattern sizes was shown to decrease the signal-to-noise ratio during recall as well. 

In contrast to palimpsest models [17-22] in which old memories are overwritten, our model assumes that all memories are equally well preserved in the synaptic states of the network, which argues for additional plasticity rules that continuously readjust the synaptic matrix to keep old memories fresh. Such a mechanism would necessarily require ongoing plasticity rules, which may then easily be linked to some sort of pattern size homeostasis that tries to keep the sparseness homogeneous. Such persistent network remodeling fits experimental findings that, at least for a few weeks after memory acquisition, existing memories can be extinct by blocking protein synthesis together with memory reactivation [23], hinting at the presence of plasticity mechanisms during early retrieval. 

## Appendix

In this appendix, we give the mathematical details of how we derive the expectation values necessary for our dynamical model from the underlying stochasticity of the recurrent synaptic matrix. The effect of learning on the synaptic connections is modeled by binary random variables that take a value $s_{ij}=1$ if the putative synapse from neuron *j* to neuron *i* is in a potentiated state (is able to transmit signals), whereas $s_{ij}=0$ if it is inactive and cannot contribute to the postsynaptic depolarization. Since not all neurons are considered to be synaptically connected, the real synaptic weight is a product $w_{ij}s_{ij}$[9] where $w_{ij}=1$ or 0 according to a binomial process with probability $c_{m}$ (the morphological connectivity) that is supposed to model the existence of a physical synapse. The two random variables $s_{ij}$ and $w_{ij}$ are considered to be independent.

The vector of pattern sizes $\phi =(f_{0},\ldots ,f_{P})$ defines how many neurons $M_{t}=f_{t}N$ fire in each pattern $\xi_{t}$. According to Willshaw’s rule, a given sequence of stored patterns, with sizes specified by ***ϕ***, uniquely defines the matrix of synaptic states $s_{ij}$: Only those synapses are potentiated for which the presynaptic neuron is active in pattern $\xi_{k}$*and* the postsynaptic neuron is active in pattern $\xi_{k+1}$ for at least one value k=0,…,P−1. In order to translate this learning rule into formulas, we have to introduce the theoretical concept of an *activation schedule*.

### A.1 Activation Schedule

Different neurons participate differently in the replay of memory sequences. Formally, each neuron is described by its activation schedule $A=\{a_{1},a_{2},\ldots ,a_{P}\}$, $a_{k}\in \{0,1\}$, which indicates in which patterns the neuron fires ($a_{k}=1$) or not ($a_{k}=0$). Since we assume the participation in a pattern to be random, the probability for a specific activation schedule is computed as

(18) $$P_{A}=\left(\prod_{k=1}^{\left|A\right|}f_{\alpha (k)}+\delta_{|A|=0}\right)\prod_{k=|A|+1}^{P}(1-f_{\alpha (k)})$$

where |A| is the number of patterns in which the neuron is active and α:{1,…,P}→{1,…,P} is a reordering of the patterns such that those in which the neuron is active have the |A| lowest indices. If |A|=0, the first factor equals 1 as indicated by the Kronecker symbol $\delta_{|A|=0}$.

The activation schedule of a neuron allows us to compute the fraction $\varsigma_{A}$ of putative activated synapses at a postsynaptic neuron with activation schedule *A* in analogy to the classical Willshaw idea,

(19) $$\varsigma_{A}=1-\left(\prod_{k=1}^{\left|A\right|}(1-f_{\alpha (k)-1})+\delta_{|A|=0}\right)$$

where the product on the right-hand side is the fraction of synapses remaining inactive after storing *P* sequential activations, corresponding to |A| learning steps.

In order to compute the mean connectivity in the network, we average over all postsynaptic neurons (i.e., activation schedules) and obtain

(20) $$\varsigma =\sum_{A}P_{A}\varsigma_{A}$$

(21) $$=\sum_{A,|A|>0}P_{A}\left(1-\prod_{k=1}^{\left|A\right|}(1-f_{\alpha (k)-1})\right)$$

(22) $$=1-P_{|A|=0}-\sum_{A,|A|>0}P_{A}\prod_{k=1}^{\left|A\right|}(1-f_{\alpha (k)-1})$$

(23) $$\begin{array}{rcl} & = & 1-\prod_{k=1}^{P}(1-f_{k})\\ & & -\sum_{A,|A|>0}\prod_{k=1}^{\left|A\right|}f_{\alpha (k)}\prod_{k=|A|+1}^{P}(1-f_{\alpha (k)})\prod_{k=1}^{\left|A\right|}(1-f_{\alpha (k)-1}) \end{array}$$

(24) $$=1-\prod_{k=1}^{P}(1-f_{k})-\sum_{A,|A|>0}\prod_{k=|A|+1}^{P}(1-f_{\alpha (k)})\prod_{k=1}^{\left|A\right|}f_{\alpha (k)}(1-f_{\alpha (k)-1})$$

(25) $$=1-\prod_{k=1}^{P}(1-f_{k})-\sum_{A,|A|>0}\prod_{k=1}^{P}(1-f_{\alpha (k)})\prod_{k=1}^{\left|A\right|}\frac{f_{\alpha (k)}(1-f_{\alpha (k)-1})}{1-f_{\alpha (k)}}$$

(26) $$=1-\prod_{k=1}^{P}(1-f_{k})-\prod_{k=1}^{P}(1-f_{k})\sum_{A,|A|>0}\prod_{k=1}^{\left|A\right|}\frac{f_{\alpha (k)}(1-f_{\alpha (k)-1})}{1-f_{\alpha (k)}}$$

(27) $$=1-\prod_{k=1}^{P}(1-f_{k})\left(1+\sum_{A,|A|>0}\prod_{k=1}^{\left|A\right|}\chi_{\alpha (k)}\right)$$

(28) $$=1-\prod_{k=1}^{P}(1-f_{k})\prod_{k=1}^{P}(1+\chi_{k})$$

(29) $$=1-\prod_{k=1}^{P}(1-f_{k})\left(1+\frac{f_{k}(1-f_{k-1})}{1-f_{k}}\right)$$

(30) $$=1-\prod_{k=1}^{P}(1-f_{k}f_{k-1})$$

where we have introduced the abbreviation $\chi_{k}=\frac{f_{k}(1-f_{k-1})}{1-f_{k}}$ and used the algebraic identity

(31) $$1+\sum_{A,|A|>0}\prod_{k=1}^{\left|A\right|}\chi_{\alpha (k)}=\prod_{k=1}^{P}(1+\chi_{k}).$$

Similarly, the second moment $E[\varsigma_{A}^{2}]$ is computed as

(32) $$E\left[\varsigma_{A}^{2}\right]=\sum_{A}P_{A}\varsigma_{A}^{2}$$

(33) $$=\sum_{A,|A|>0}P_{A}{\left(1-\prod_{k=1}^{\left|A\right|}(1-f_{\alpha (k)-1})\right)}^{2}$$

(34) $$=\sum_{A,|A|>0}P_{A}\left(1-2\prod_{k=1}^{\left|A\right|}(1-f_{\alpha (k)-1})+\prod_{k=1}^{\left|A\right|}{\left(1-f_{\alpha (k)-1}\right)}^{2}\right)$$

(35) $$=2\varsigma -1+\prod_{k=1}^{P}(1-f_{k})+\sum_{A,|A|>0}P_{A}\prod_{k=1}^{\left|A\right|}{\left(1-f_{\alpha (k)-1}\right)}^{2}$$

(36) $$\begin{array}{rcl} & = & 2\varsigma -1+\prod_{k=1}^{P}(1-f_{k})\\ & & +\sum_{A,|A|>0}\prod_{k=1}^{\left|A\right|}f_{\alpha (k)}\prod_{k=|A|+1}^{P}(1-f_{\alpha (k)})\prod_{k=1}^{\left|A\right|}{\left(1-f_{\alpha (k)-1}\right)}^{2} \end{array}$$

(37) $$\begin{array}{rcl} & = & 2\varsigma -1+\prod_{k=1}^{P}(1-f_{k})\\ & & +\sum_{A,|A|>0}\prod_{k=|A|+1}^{P}(1-f_{\alpha (k)})\prod_{k=1}^{\left|A\right|}f_{\alpha (k)}{\left(1-f_{\alpha (k)-1}\right)}^{2} \end{array}$$

(38) $$\begin{array}{rcl} & = & 2\varsigma -1+\prod_{k=1}^{P}(1-f_{k})\\ & & +\sum_{A,|A|>0}\prod_{k=1}^{P}(1-f_{\alpha (k)})\prod_{k=1}^{\left|A\right|}\frac{f_{\alpha (k)}{\left(1-f_{\alpha (k)-1}\right)}^{2}}{1-f_{\alpha (k)}} \end{array}$$

(39) $$\begin{array}{rcl} & = & 2\varsigma -1+\prod_{k=1}^{P}(1-f_{k})\\ & & +\prod_{k=1}^{P}(1-f_{k})\sum_{A,|A|>0}\prod_{k=1}^{\left|A\right|}\frac{f_{\alpha (k)}{\left(1-f_{\alpha (k)-1}\right)}^{2}}{1-f_{\alpha (k)}} \end{array}$$

(40) $$=2\varsigma -1+\prod_{k=1}^{P}(1-f_{k})\prod_{k=1}^{P}\left(1+\frac{f_{k}{\left(1-f_{k-1}\right)}^{2}}{1-f_{k}}\right)$$

(41) $$=2\varsigma -1+\prod_{k=1}^{P}\left(1-f_{k}+f_{k}{\left(1-f_{k-1}\right)}^{2}\right)$$

(42) $$=2\varsigma -1+\prod_{k=1}^{P}\left(1-f_{k}\left(2f_{k-1}-f_{k-1}^{2}\right)\right).$$

### A.2 Mean and Variance of Total Synaptic Input

With the above two moments, we can find means and variances for the synaptic inputs. We start with the probability of total synaptic input to a postsynaptic cell with activation schedule *A*, which is binomially distributed according to

(43) P(h|A)=(m+nh)(cmςA)h(1−cmςA)(m+n−h).

The probability of total synaptic input to an average postsynaptic cell can then be obtained as

(44) $$P(h)=\sum_{A}P_{A}P(h|A).$$

The mean value of *h* depends on whether the postsynaptic cell belongs to the **On** population (should fire) or the **Off** population (should not fire). For the **Off** population, we have

(45) $$\mu_{\mathrm{Off}}=\sum_{h=0}^{m+n}hP(h)$$

(46) $$=\sum_{h=0}^{m+n}h\sum_{A}P_{A}P(h|A)$$

(47) $$=\sum_{A}P_{A}\sum_{h=0}^{m+n}hP(h|A)$$

(48) =∑APA∑h=0m+nh(m+nh)(cmςA)h(1−cmςA)(m+n−h)

(49) $$=\sum_{A}P_{A}(m+n)c_{m}\varsigma_{A}$$

(50) $$=c_{m}(m+n)\sum_{A}P_{A}\varsigma_{A}$$

(51) $$=c_{m}\varsigma (m+n)$$

and for the **On** population,

(52) $$\mu_{\mathrm{On}}=\sum_{h^{\prime }=0}^{m}h^{\prime }P\left(h^{\prime }\right)+\sum_{h^{\prime \prime }=0}^{n}h^{\prime \prime }P\left(h^{\prime \prime }\right)$$

(53) $$=c_{m}m+c_{m}\varsigma n.$$

In order to obtain the variance of *h*, we compute the second moment of *h* for the **Off** population,

(54) $$E\left[h_{\mathrm{Off}}^{2}\right]=\sum_{h=0}^{m+n}h^{2}P(h)$$

(55) =∑APA∑h=0m+nh2(m+nh)(cmςA)h(1−cmςA)(m+n−h)

(56) $$=\sum_{A}P_{A}(m+n)c_{m}\varsigma_{A}\left(1+c_{m}\varsigma_{A}(m+n-1)\right)$$

(57) $$=c_{m}(m+n)\sum_{A}P_{A}\varsigma_{A}\left(1+c_{m}\varsigma_{A}(m+n-1)\right)$$

(58) $$=c_{m}(m+n)\left(\sum_{A}P_{A}\varsigma_{A}+\sum_{A}P_{A}\varsigma_{A}^{2}c_{m}(m+n-1)\right)$$

(59) $$=c_{m}(m+n)\left(\varsigma +E\left[\varsigma_{A}^{2}\right]c_{m}(m+n-1)\right).$$

The variance is then given by

(60) $$\sigma_{\mathrm{Off}}^{2}=E\left[h_{\mathrm{Off}}^{2}\right]-\mu_{\mathrm{Off}}^{2}$$

(61) $$=c_{m}(m+n)\left(\varsigma +E\left[\varsigma_{A}^{2}\right]c_{m}(m+n-1)\right)-{\left(c_{m}\varsigma (m+n)\right)}^{2}$$

(62) $$=c_{m}\varsigma (m+n)\left(1+\frac{E[\varsigma_{A}^{2}]}{\varsigma }c_{m}(m+n-1)-c_{m}\varsigma (m+n)\right)$$

(63) $$=c_{m}\varsigma (m+n)\left(1-c_{m}\varsigma +\frac{E[\varsigma_{A}^{2}]-\varsigma^{2}}{\varsigma }c_{m}(m+n-1)\right)$$

(64) $$=c_{m}\varsigma (m+n)\left(1-c_{m}\varsigma +V_{\varsigma }^{2}c_{m}\varsigma (m+n-1)\right)$$

where

(65) $$V_{\varsigma }^{2}=\frac{E[\varsigma_{A}^{2}]-\varsigma^{2}}{\varsigma^{2}}=\frac{1}{\varsigma^{2}}\left(2\varsigma -1+\prod_{k=1}^{P}\left(1-f_{k}\left(2f_{k-1}-f_{k-1}^{2}\right)\right)\right)-1$$

is the squared variation coefficient of $\varsigma_{A}$ over all activation schedules *A*. Similarly, for the **On** population, we get

(66) $$\sigma_{\mathrm{On}}^{2}=c_{m}m(1-c_{m})+c_{m}\varsigma n\left(1-c_{m}\varsigma +V_{\varsigma }^{2}c_{m}\varsigma (n-1)\right).$$

### A.3 Mean and Variance of *ς* over Pattern Size Distribution

So far, all formulas were obtained for a specific realization of the pattern size (coding ratio) vector ***ϕ***. The pattern sizes themselves can, however, be considered as resulting from a stochastic process as well. We therefore are interested in expectation values over the pattern size distribution to be able to account for average connectivities over many realizations of the network. Such an average connectivity 〈ς〉 upon imprinting the memory with *P* patterns is given by

(67) $$\langle \varsigma \rangle =1-\langle \prod_{k=1}^{P}(1-f_{k}f_{k-1})\rangle$$

where 〈⋅〉 indicates the expected value over the size distribution $p_{\phi }(\phi )$. The last term can be expanded as follows:

(68) $$\langle \prod_{t=1}^{P}(1-f_{t}f_{t+1})\rangle =1-\sum_{t=1}^{P}\langle f_{t}f_{t+1}\rangle +\sum_{t=1}^{P}\sum_{t^{\prime }=t+1}^{P}\langle f_{t}f_{t+1}f_{t^{\prime }}f_{t^{\prime }+1}\rangle -\cdots$$

The last term on the right, as well as higher-order terms, contain overlapping indices (e.g., when $t^{\prime }=t+1$), so that for each term of order 2*k* (for k=1,…,P), we will have 2*j* isolated indices, each contributing a term 〈f〉, and (k−j) duplicated indices, each contributing a term $\langle f^{2}\rangle$ (for j=1,…,k). Therefore, we can write

(69) $$\langle \prod_{t=1}^{P}(1-f_{t}f_{t+1})\rangle =1+\sum_{k=1}^{P}{\left(-1\right)}^{k}\left(\sum_{j=1}^{k}n_{j}^{\left(P,k\right)}{\langle f\rangle }^{2j}{\langle f^{2}\rangle }^{k-j}\right)$$

where $n_{j}^{\left(P,k\right)}$ is the number of *k*-combinations of *P* elements with exactly *j* non-adjacent element sets. For example, given *P* elements $a_{1},a_{2},\ldots ,a_{P}$, the 3-combination $a_{1}a_{2}a_{3}$ has j=1 non-adjacent sets, $a_{1}a_{3}a_{4}$ has j=2, and $a_{1}a_{3}a_{5}$ has j=3. After some algebra, we arrive at the expression

(70) nj(P,k)={(P−k+1j)(k−1k−j),j≤min(k,P−k+1),0,otherwise.

The mean probability of synaptic potentiation 〈ς〉 can thus be expressed as a function of the first and second order moments of the coding ratio distribution $p_{\phi }(\phi )$ as follows:

(71) $$\langle \varsigma \rangle =-\sum_{k=1}^{P}\sum_{j=1}^{k}{\left(-1\right)}^{k}n_{j}^{\left(P,k\right)}{\langle f\rangle }^{2j}{\langle f^{2}\rangle }^{k-j}$$

with the first and second moments

(72) $$\langle f\rangle =\phi_{0},$$

(73) $$\langle f^{2}\rangle =\sigma_{\phi }^{2}+\phi_{0}^{2}.$$

The second moment $\langle \varsigma^{2}\rangle$ of the probability of synaptic potentiation is given by

(74) $$\langle \varsigma^{2}\rangle =\langle {\left(1-\prod_{k=1}^{P}(1-f_{k}f_{k-1})\right)}^{2}\rangle$$

(75) $$=1-2\langle \prod_{k=1}^{P}(1-f_{k}f_{k-1})\rangle +\langle \prod_{k=1}^{P}{\left(1-f_{k}f_{k-1}\right)}^{2}\rangle$$

(76) $$=2\langle \varsigma \rangle -1+\langle \prod_{k=1}^{P}{\left(1-f_{k}f_{k-1}\right)}^{2}\rangle$$

where the last term equals

(77) $$\langle \prod_{t=1}^{P}{\left(1-f_{t}f_{t+1}\right)}^{2}\rangle$$

(78) $$\begin{array}{rl} & \;=1-\sum_{t=1}^{P}\langle \left(2f_{t}f_{t+1}-f_{t}^{2}f_{t+1}^{2}\right)\rangle \\ & \;+\sum_{t=1}^{P}\sum_{t^{\prime }=t+1}^{P}\langle \left(2f_{t}f_{t+1}-f_{t}^{2}f_{t+1}^{2}\right)\left(2f_{t^{\prime }}f_{t^{\prime }+1}-f_{t^{\prime }}^{2}f_{t^{\prime }+1}^{2}\right)\rangle -\cdots \end{array}$$

(79) =1+∑k=1P∑j=1k(−1)k(P−k+1j)Skj.

Here, we use

(80) $$S_{kj}=\left\{ \begin{array}{ll} \pi_{k}, & j=1,\\ \sum_{i=1}^{k-j+1}\pi_{i}S_{k-i,j-1}, & 2\leq j\leq k \end{array}\right.$$

and

(81) $$\pi_{k}=\langle \prod_{t=1}^{k}\left(2f_{t}f_{t+1}-f_{t}^{2}f_{t+1}^{2}\right)\rangle$$

(82) $$=\sum_{m=0}^{k}{\left(-1\right)}^{m}2^{k-m}\psi_{m}$$

with

(83) ψm={〈f〉2〈f2〉k−1,m=0,〈f〉2∑i=1mk−m−ik−mni(k−1,m)〈f2〉k−m−i−1〈f3〉2i〈f4〉m−i+2〈f〉〈f2〉∑i=1mik−mni(k−1,m)〈f2〉k−m−i〈f3〉2i−1〈f4〉m−i+〈f2〉2∑i=1k−mm−imni(k−1,k−m)〈f2〉k−m−i〈f3〉2i〈f4〉m−i−1,〈f2〉2〈f4〉k−1,1≤m≤k−1,〈f2〉2〈f4〉k−1,m=k.

If $p_{\phi }(\phi )$ is the Gamma distribution, the higher moments can be computed as

(84) $$\langle f^{3}\rangle =2\sigma_{\phi }^{4}/\phi_{0}+3\sigma_{\phi }^{2}\phi_{0}+\phi_{0}^{3},$$

(85) $$\langle f^{4}\rangle =6\sigma_{\phi }^{6}/\phi_{0}^{2}+8\sigma_{\phi }^{4}+6\sigma_{\phi }^{2}\phi_{0}^{2}+\phi_{0}^{4}.$$

Finally, the variance $\sigma_{\varsigma }^{2}$ of the probability of synaptic potentiation over all possible realizations of the coding ratio vector ***ϕ*** is given by

(86) $$\sigma_{\varsigma }^{2}=\langle \varsigma^{2}\rangle -{\langle \varsigma \rangle }^{2}.$$

## Competing Interests

The authors declare that they have no competing interests.

## Authors’ Contributions

DM and CL performed the mathematical analysis. DM carried out the computer simulations. DM and CL drafted the manuscript.
